# Pathways between objective and perceived neighborhood factors among Black breast cancer survivors

**DOI:** 10.1186/s12889-021-12057-0

**Published:** 2021-11-06

**Authors:** Jesse J. Plascak, Adana A. M. Llanos, Stephen J. Mooney, Andrew G. Rundle, Bo Qin, Yong Lin, Karen S. Pawlish, Chi-Chen Hong, Kitaw Demissie, Elisa V. Bandera

**Affiliations:** 1 Comprehensive Cancer Center, The Ohio State University, Columbus, OH USA; 2 Division of Cancer Prevention and Control, Department of Internal Medicine, College of Medicine, The Ohio State University, 1590 North High Street, Suite 525, Columbus, OH 43201 USA; 3 Cancer Prevention and Control Program, Rutgers Cancer Institute of New Jersey, New Brunswick, NJ USA; 4 Department of Epidemiology, Mailman School of Public Health, Columbia University, New York, NY USA; 5 Department of Epidemiology, School of Public Health, University of Washington, Seattle, Washington USA; 6 Department of Biostatistics and Epidemiology, School of Public Health, Rutgers, The State University of New Jersey, Piscataway, NJ USA; 7 New Jersey State Cancer Registry, New Jersey Department of Health, Trenton, NJ USA; 8 Department of Cancer Prevention and Control, Roswell Park Cancer Institute, Buffalo, New York USA; 9 Department of Epidemiology and Biostatistics, School of Public Health, SUNY Downstate Health Sciences University, Brooklyn, NY USA

**Keywords:** Objective neighborhood disorder, Perceived neighborhood disorder, Perceived neighborhood social cohesion, Breast cancer survivors

## Abstract

**Background:**

Mounting evidence supports associations between objective neighborhood disorder, perceived neighborhood disorder, and health, yet alternative explanations involving socioeconomic and neighborhood social cohesion have been understudied. We tested pathways between objective and perceived neighborhood disorder, perceived neighborhood social cohesion, and socioeconomic factors within a longitudinal cohort.

**Methods:**

Demographic and socioeconomic information before diagnosis was obtained at interviews conducted approximately 10 months post-diagnosis from participants in the Women’s Circle of Health Follow-up Study – a cohort of breast cancer survivors self-identifying as African American or Black women (*n* = 310). Neighborhood perceptions were obtained during follow-up interviews conducted approximately 24 months after diagnosis. Objective neighborhood disorder was from 9 items audited across 23,276 locations using Google Street View and scored to estimate disorder values at each participant’s residential address at diagnosis. Census tract socioeconomic and demographic composition covariates were from the 2010 U.S. Census and American Community Survey. Pathways to perceived neighborhood disorder were built using structural equation modelling. Model fit was assessed from the comparative fit index and root mean square error approximation and associations were reported as standardized coefficients and 95% confidence intervals.

**Results:**

Higher perceived neighborhood disorder was associated with higher objective neighborhood disorder (β = 0.20, 95% CI: 0.06, 0.33), lower neighborhood social cohesion, and lower individual-level socioeconomic factors (final model root mean square error approximation 0.043 (90% CI: 0.013, 0.068)). Perceived neighborhood social cohesion was associated with individual-level socioeconomic factors and objective neighborhood disorder (β = − 0.11, 95% CI: − 0.24, 0.02).

**Conclusion:**

Objective neighborhood disorder might be related to perceived disorder directly and indirectly through perceptions of neighborhood social cohesion.

## Background

The proliferation of public health studies of ‘neighborhood factors’ has led to a multitude of constructs potentially involved in pathways between social and built environments and health. For example, reviews of ‘neighborhood disorder‘, ‘social capital’, ‘neighborhood disadvantage’, and ‘residential segregation’ have appeared in public health journals since 2017 [1–4]. Many of these reviews provide evidence for associations between adverse neighborhood factors and poorer health, and that numerous sub-measures exist within each broader construct (e.g., observed and perceived versions of both physical and social neighborhood disorder). Despite abundant literature on relationships between various urban neighborhood characteristics and health behaviors and outcomes, few studies have investigated pathways between various neighborhood measures. This is a critical gap because social and built environmental factors are inter-related [2, 5]. Knowledge of relationships among various neighborhood factors is especially important to urban health for several reasons, including: quantifying magnitudes of association to inform relative influence of factors, motivating data collection protocols of future studies, understanding relevant pathways and mechanisms to health, and identifying points of intervention.

Social cohesion, social disorder, and physical disorder are commonly studied neighborhood factors [1, 2]. Perceived neighborhood social cohesion measures the level of trust and shared values among community members [1]. Social disorder refers to the breakdown of social relationships within a neighborhood and is indicated by norm-breaking activities such as crime, loitering, public substance use, and inter-personal conflict [6, 7]. Physical disorder has previously been conceptualized as a byproduct of deteriorating social norms and relationships and indicated by excessive garbage, graffiti, abandoned buildings, loud noises, and noxious odors [6, 7]. Perceptions of less social cohesion may be both a precursor to and effect of neighborhood disorder (perceived and objective); lower trust and shared values among neighbors could lead to a breakdown of social relationships and investments in the physical spaces within a neighborhood [8], or neighborhoods marked by higher disorder could lead to reduced social cohesion among neighbors [9, 10].

**Fig. 1:**
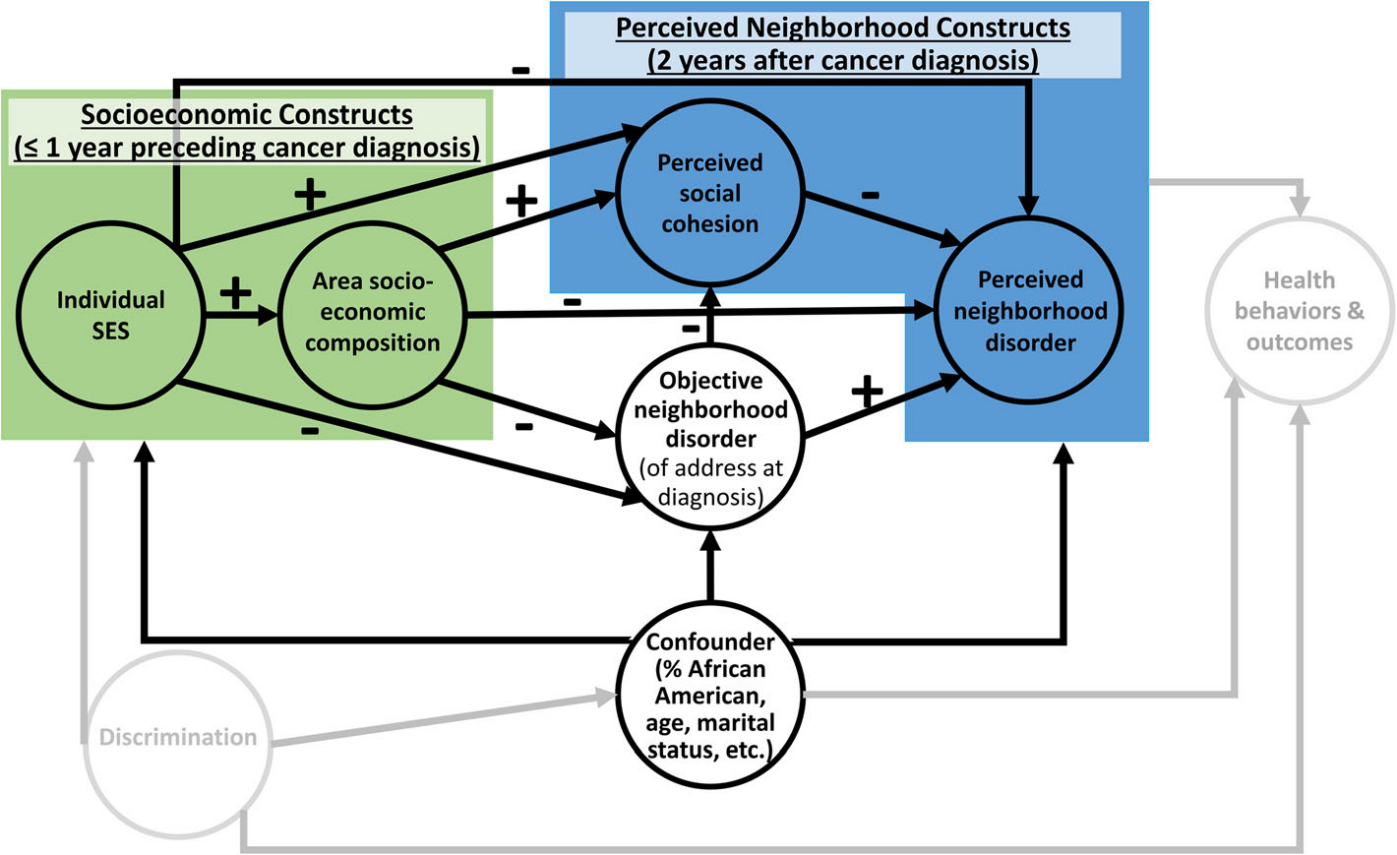
Hypothesized associations between health-related neighborhood factors, socioeconomic factors, and potential confounders^1,2^. ^1^ Shaded figures that are less visible are unmeasured in this study, but displayed to provide a more complete description of relationships. ^2^ Individual-level potential confounders were assessed within the year preceding a participant’s breast cancer diagnosis (2013–2018) and census tract-level potential confounders were calculated from 2010 decennial census data linked to residential address at diagnosis

Recent, randomized trials have drawn attention to the role of objective neighborhood disorder as experimental vacant lot remediation, garbage and graffiti removal, and structural maintenance were associated with lower perceptions of nearby crime, lower objective measures of crime, and improvements in self-reported mental health [11, 12]. A recent meta-analysis of observational studies supports these results and concludes that an individual’s perception of neighborhood disorder might serve as the mediator between objective disorder and health behaviors and outcomes [2]. This same systematic review of neighborhood disorder also concludes that perceived neighborhood social cohesion and socioeconomic factors are understudied, despite early evidence that such factors influence perceived and objective measures of disorder [6, 13, 14]. Neighborhood socioeconomic disadvantage and individual socioeconomic factors are particularly important for their potential explanation of disparities by marginalized identities and oppressed people (African American/Black population in the U.S., women, older populations, etc.) due to social stratification caused by structural discrimination (e.g., racial-ethnic housing discrimination, occupational ageism and sexism) [15] (Fig. 1).

Despite experimental and meta-analysis evidence supporting relationships with health outcomes, studies have criticized the potential role that objective neighborhood disorder might play [2, 6, 13]. This de-emphasis might be due to assertions that objective physical disorder, as a measure of broken windows theory, has contributed to over-policing communities of color [16, 17], as well as empirical evidence for attenuated associations between objective and perceived neighborhood disorder with adjustment for sociodemographic covariates (i.e., gender, individual-level socioeconomic factors, neighborhood racial-ethnic composition) and neighborhood social cohesion or collective efficacy [6, 13, 14]. However, more recent evidence supports conceptualization of observed neighborhood disorder as a product of discrimination and disinvestment of individuals and communities of color [18, 19]. Moreover, many empirical studies of perceived neighborhood disorder that investigated the roles of socioeconomic factors, perceived neighborhood social cohesion, and observed neighborhood disorder have been cross-sectional, leaving the possibility of unclear directionality of associations [6, 13, 14].

Various socio-ecologic frameworks have been developed to better understand the potential interrelatedness of neighborhood constructs and their involvement within pathways to adverse health outcomes [20, 21], including breast cancer [5, 22]. We draw on these frameworks to position present-day, observed neighborhood disorder as a direct product of current and historical systemic racism [15]. Institutional racism within mortgage lending practices and policies (i.e., redlining and reverse redlining) has been linked to both lower individual-level socioeconomic well-being, greater racial-ethnic segregation, and worse neighborhood conditions [23, 24], with particularly adverse effects among racially-ethnically marginalized communities [18, 25–28]. Persistent racial-ethnic and socioeconomic stratification has led to racially-ethnically segregated social networks, and in turn, residential mobility patterns such that individual racial-ethnic identity and socioeconomic status influence neighborhood-level racial-ethnic and socioeconomic composition [29, 30]. Similar racism-based measures of lending discrimination and observed neighborhood disorder have been linked to adverse breast cancer outcomes [31–33], which disproportionately affect Latinx and Black women [34–36].

Knowledge of these relationships is especially important among Black breast cancer survivors; a growing population within which few studies of objective and perceived neighborhood factors have been conducted [34, 37]. Despite residence in areas of higher objective neighborhood disorder [33, 38], Black survey respondents tend to perceive less disorder [6, 13]. In contrast, women tend to report higher levels neighborhood disorder than men, even after accounting for objective measures of neighborhood disorder [6, 13]. Various objective neighborhood factors could impact breast cancer-related behaviors and outcomes through perceived disorder [2, 22], but no studies of such neighborhood factors have been conducted among Black breast cancer survivors. For example, a meta-analysis of neighborhood disorder and health behaviors supported associations between greater substance use, lower physical activity, and higher perceived disorder – risk factors associated with worse survival outcomes among breast cancer cases [39, 40]. Moreover, few studies have measured time-sequenced pathways involving socioeconomic factors, observed neighborhood disorder, and perceived neighborhood characteristics among any population, preventing robust interpretations of relationships between these inter-related neighborhood factors. The purpose of this study is to test pathways between objective and perceived neighborhood disorder, perceived neighborhood social cohesion, and socioeconomic factors among a longitudinal cohort of Black breast cancer survivors (Fig. 1).

## Methods

### Study population and data

Individual-level data were from the Women’s Circle of Health Follow-up Study (WCHFS), a cohort of Black breast cancer survivor cohort built upon the infrastructure of the Women’s Circle of Health Study. The design and methods – including questionnaire elements – of the WCHFS has been described in detail elsewhere [41]. In brief, women self-identifying as Black or African American, with a recent diagnosis of breast cancer between the years 2013 and 2018 and no prior history of cancer (except non-melanoma skin cancer), residing within a 10-county region of New Jersey at time of diagnosis, between 20 and 75 years of age, and able to speak and understand English were eligible for the study. We additionally restricted to participants residing in urban areas (Rural-Urban Commuting Area < 2/‘Metropolitan Core’) because neighborhood disorder is considered an urban construct [6, 7, 14], and we limited to the most accurate geocodes (full street address and zip code) to reduce measurement error of geographic-based factors. Potential participants were contacted by New Jersey State Cancer Registry staff to determine eligibility and interest in the study. Eligible and interested women were then contacted by study personnel to provide further study details and to schedule an in-person interview. Women who provided written informed consent during the in-person interview were administered a baseline questionnaire of various relevant items, including demographic and socioeconomic factors. Participants were asked to report their marital status, educational attainment, household income, and number of individuals supported by that income as they were 1 year prior to cancer diagnosis (approximately 21 months prior to baseline survey). Participants were also asked about their birth country. Age, geocoded residential address, and geocoding accuracy were from the New Jersey State Cancer Registry as assessed at date of breast cancer diagnosis.

Neighborhood perception items were included within an in-person, follow-up questionnaire that was administered approximately 2 years following diagnosis (15 months following baseline questionnaire). Current, perceived neighborhood disorder was a 13-item scale and neighborhood social cohesion was a 5-item scale [7, 8]. The social and physical subscales of perceived neighborhood disorder comprised 7 and 6 items, respectively. Social disorder items focus on perceptions of loitering, crime, drug and alcohol use, neighbor conflict, neighborly care for one another, and neighborhood safety, while physical disorder items focus on graffiti, noise, vandalism, abandoned buildings, cleanliness, and home maintenance within the neighborhood. Item responses were five-level Likert scales of agreement ranging from ‘strongly disagree’ to ‘strongly agree’. Separate indices of social and physical disorder were created upon finding adequate internal consistency reliability (Cronbach α_social disorder_ = 0.862, Cronbach α_physical disorder_ = 0.821). Non-missing physical disorder item responses were sum scored. Only one social disorder item was unanswered by 6% of participants (8% missing responses on 2 or more items). Thus social disorder item responses were mean scored if no more than one item was missing. Perceived neighborhood social cohesion responses used the same agreement scale on the following items: neighbors’ willingness to help each other, neighbors being trustworthy, neighbors getting along, neighbors sharing identical values, and neighborhood being close-knit. Similar to perceived social disorder, mean scoring was used to create an index of perceived social cohesion as 10% of participants were missing a response to only one item (Cronbach α_social cohesion_ = 0.806). Scores were proportional to greater disorder or cohesion and theoretical ranges were as follows: 1–5 social cohesion, 1–5 social disorder, 6–30 physical disorder. Duration in current neighborhood (< 2 years, 2–4 years, > 4 years) was also from the follow-up questionnaire.

Census tract-level, neighborhood factors were based on 2010 U.S. Census data and the American Community Survey [42]. Neighborhood socioeconomic composition was from previously calculated, annual indices based on factor analyses of decennial census and American Community Survey, 5-year estimates of percent working class, percent aged at least 16 years who are unemployed, percent of persons below 150% of the federal poverty line, median household income, an education index (weighted school years), median house value, and median rent [43]. Neighborhood socioeconomic composition data were provided as vigintiles (20 levels) for years 2000–2015 and linked to participant’s year of diagnosis [44]. Women diagnosed after 2015 were assigned neighborhood socioeconomic composition data from 2015. Census tract African American/Black composition, population density, and African American/Black residential segregation indices (Gini and Isolation) were from 2010 decennial data. Population density (per square kilometer (km)) was from 2010 Census data and census tract area calculated within a geographic information system.

Baseline covariates considered in analyses were handled as follows: age (continuous years), birth country (categorical, non-U.S. vs U.S.), marital status (nominal, married or living as married; separated, divorced, or widowed; single and never married), educational attainment (ordinal levels, < 8th grade, 8th – 11th grade, high school graduate or equivalent, technical or vocational school, some college, college graduate, post-graduate), household income-to-poverty ratio (continuous values based on household income, household size, and annual federal poverty levels) [45].

Objective neighborhood disorder was assessed throughout the study region using a virtual audit platform that interfaces with Google Street View scenes [46]. Audit protocol, item measurement properties, construct creation, and participant-level prediction of physical disorder-related constructs have been previously described [38, 47]. Briefly, nine items related to neighborhood disorder – garbage/litter (present/absent), graffiti (present/absent), boarded/burned buildings (present/absent), dumpsters (none, < 2, ≥2), building conditions (very well kept, moderately well kept, fairly well kept, poorly well kept), yard conditions (very well kept, moderately well kept, fairly well kept, poorly well kept), team sports equipment in public spaces (present/absent), yard decorations (present/absent), and outdoor seating (present/absent) – were assessed at 23,276 locations across urban census tracts. Item response theory of the nine items resulted in a model best fit by two factors: ‘physical disorder’ indicated by garbage/litter, graffiti, boarded/burned buildings, dumpsters, building conditions, and yard conditions loading; and ‘engagement’ indicated by team sports equipment in public spaces (present/absent), yard decorations (present/absent), and outdoor seating. While objective physical disorder has been similarly assessed in previous studies [2, 48], indicators of engagement are less commonly studied but might be associated with neighborhood social norms [38, 49]. Universal Local Kriging of item response theory factor scores yielded address-level estimates of physical disorder and engagement for each participant [38, 48]. Kriging analyses indicated strong spatial autocorrelation patterns in physical disorder (nugget = 0.49, partial sill = 0.32, range = 8.2 km) and weak spatial autocorrelation of engagement (nugget = 0.28, partial sill = 0.03, range = 2.5 km). Higher scores were proportional to more physical disorder and engagement.

### Statistical analysis

Descriptive statistics (means and standard deviations for continuous variables, frequencies and percentages for categorical variables) of each covariate were calculated. Perceived disorder and population density distributions were right-skewed; we log-transformed both measures prior to correlation analyses. Bi-variate relationships between variables were examined through Pearson correlation coefficients. Structural equation modelling was used to estimate standardized coefficients and 95% confidence intervals (CI) from a final model involving variables as depicted in Fig. 1 [50]. Three models were built with the goal of testing hypothesized and best-fitting alternative models: model 1 - the hypothesized model excluding confounders (Fig. 1), model 2 - eliminate poorly fitting pathways of model 1 (i.e., wide 95% CI), and model 3 - expand model 2 to include covariates associated with combinations of socioeconomic or neighborhood variables (i.e., confounders). Latent constructs were indicated by variables as follows: individual socioeconomic status indicated by educational attainment and income-to-poverty ratio; objective neighborhood disorder indicated by audited physical disorder and engagement; and perceived neighborhood disorder indicated by perceived social and physical disorder. We report comparative fit index and root mean square error approximation model fit measures and base conclusions of a good fitting final model if comparative fit index (comparing a null model) was greater than 0.90 and the upper bound of the 90% CI of root mean square error approximation was less than 0.08 [50]. We report overall model fit with the best fitting model having a lower root mean square error approximation. Standardized coefficients are only reported of the final, best model. Model residuals were examined for outliers. As a post-hoc sensitivity analysis, we restricted the final model to those who did not move neighborhoods within the previous 2 years.

The analytic dataset was restricted to non-missing variable values, with missingness as follows: *n* = 41 (9.7%) income, *n* = 2 (0.5%) marital status, *n* = 1 (0.2%) education, n = 1 (0.2%) duration of residence in neighborhood, *n* = 44 (10.4%) social cohesion, *n* = 16 (3.8%) perceived physical disorder, *n* = 36 (8.5%) perceived social disorder, and *n* = 10 (2.3%) neighborhood socioeconomic composition. Of 421 eligible participants with follow-up data, 310 had non-missing data. Missing data analyses indicated nearly identical distributions of covariates when comparing results across datasets that included missing values. WCHFS data were gathered between 2013 to 2020. Audit data were collected November 2017 to June 2019.

## Results

Table 1 displays participant characteristics. Approximately 30% of participants were single and never married, average household income was less than 3 times the federal poverty level, 33% of participants had a high school education or less, and 86% were born in the US. Participants resided in census tracts of moderate-to-high African American/Black residential segregation as indicated by average Gini, Isolation, and African American/Black composition values of 56.5, 52.2, and 45.2%, respectively. As expected in New Jersey – the most densely populated US state– population density in this cohort was high (mean 4471 people/km^2^)

**Table 1:** Descriptive statistics of demographic, socioeconomic, perceived neighborhood, objective neighborhood, and census-based neighborhood factors, Women’s Circle of Health Follow-up Study, *n* = 310

Variable	Mean/N (SD/%)
Age at diagnosis, yr	56.0 (10.4)
Marital status	
Married/living as married	109 (35.2)
Separated/divorced/widowed	106 (34.2)
Single/never married	95 (30.6)
Income-to-poverty ratio	2.8 (2.0)
Educational attainment	
≤ High school	102 (32.9)
Technical school/some college	108 (34.8)
College graduate	63 (20.3)
Post-graduate	37 (11.9)
U.S. born	266 (85.8)
Duration in neighborhood	
< 2 years	21 (6.8)
2–4 years	61 (19.7)
> 4 years	228 (73.5)
Neighborhood perceptions	
Social cohesion (mean scored)	3.6 (0.92)
Social disorder (mean scored)	1.8 (0.89)
Physical disorder (sum scored)	10.1 (4.8)
Neighborhood audits	
Engagement	0.02 (0.24)
Physical disorder	0.44 (0.58)
Census tract-level	
Socioeconomic composition (vigintile)	9.3 (5.3)
African American segregation, Gini	56.5 (14.9)
African American segregation, Isolation	52.2 (26.3)
African American composition	45.2 (29.6)
Population density, ppl per km^2^	4471 (3440)

Pearson correlations between measured variables were generally higher among the perception-, audit-, and census-based neighborhood variables (Table 2). Perceived social and physical disorder were strongly correlated (Pearson *r* = 0.8). As such, both perceived social and.

**Table 2:** Pearson correlations between demographic, socioeconomic, perceived neighborhood, objective neighborhood, and census-based neighborhood factors, Women’s Circle of Health Follow-up Study, *n* = 310^1^

	1.	2.	3.	4.	5.	6.	7.	8.	9.	10.	11.	12.	13.	14.	15.
1.Age															
2.Married/long-term relationship	−.031														
3.Income-to-poverty ratio	.115	.227													
4.Education	−.028	.190	.459												
5.U.S. born	.125	−.184	−.022	−.028											
6.Neighborhood residence > 4 years	.180	.273	.128	.024	.070										
7.Perceived neighborhood social cohesion	.177	.093	.171	.176	.021	.057									
8.Perceived neighborhood social disorder^2^	−.087	−.208	−.268	−.318	.167	−.040	−.451								
9.Perceived neighborhood physical disorder^2^	−.115	−.194	−.265	−.323	.137	−.058	−.526	.780							
10.Objective neighborhood physical disorder	−.043	−.196	−.273	−.208	.174	−.113	−.276	.498	.417						
11.Objective neighborhood engagement	−.048	.016	.011	−.005	.056	.044	.023	−.150	−.095	−.162					
12.Neighborhood socioeconomic composition	.039	.232	.299	.318	−.174	.068	.246	−.469	−.411	−.681	.063				
13.African American Gini segregation	.003	.051	−.197	−.108	−.092	.006	.027	.064	.065	−.006	−.030	.015			
14.African American Isolation segregation	.023	−.042	−.098	−.092	.132	−.028	−.144	.254	.237	.500	−.052	−.504	−.073		
15.Census tract African American composition	.012	−.043	−.077	−.058	.116	−.023	−.155	.252	.237	.507	−.078	−.501	−.172	.980	
16.Population density^2^	.005	−.092	−.193	−.157	.132	.014	−.314	.476	.425	.538	−.173	−.594	−.114	.394	.412

^1^ *p*-value ≤0.05 among correlations ≥ |0.113|

^2^ Log-transformed

physical disorder were similarly correlated with other variables as follows: weakly-to-moderately inversely correlated with education and income-to-poverty ratio, moderately inversely correlated with social cohesion, moderately positively correlated with objective physical disorder, and moderately positively correlated with population density. Objective physical disorder was weakly inversely correlated with education, income-to-poverty ratio, and social cohesion; and moderately-to-strongly inversely correlated with census tract socioeconomic composition, African American/Black isolation index, African American/Black composition, and population density.

**Fig. 2:**
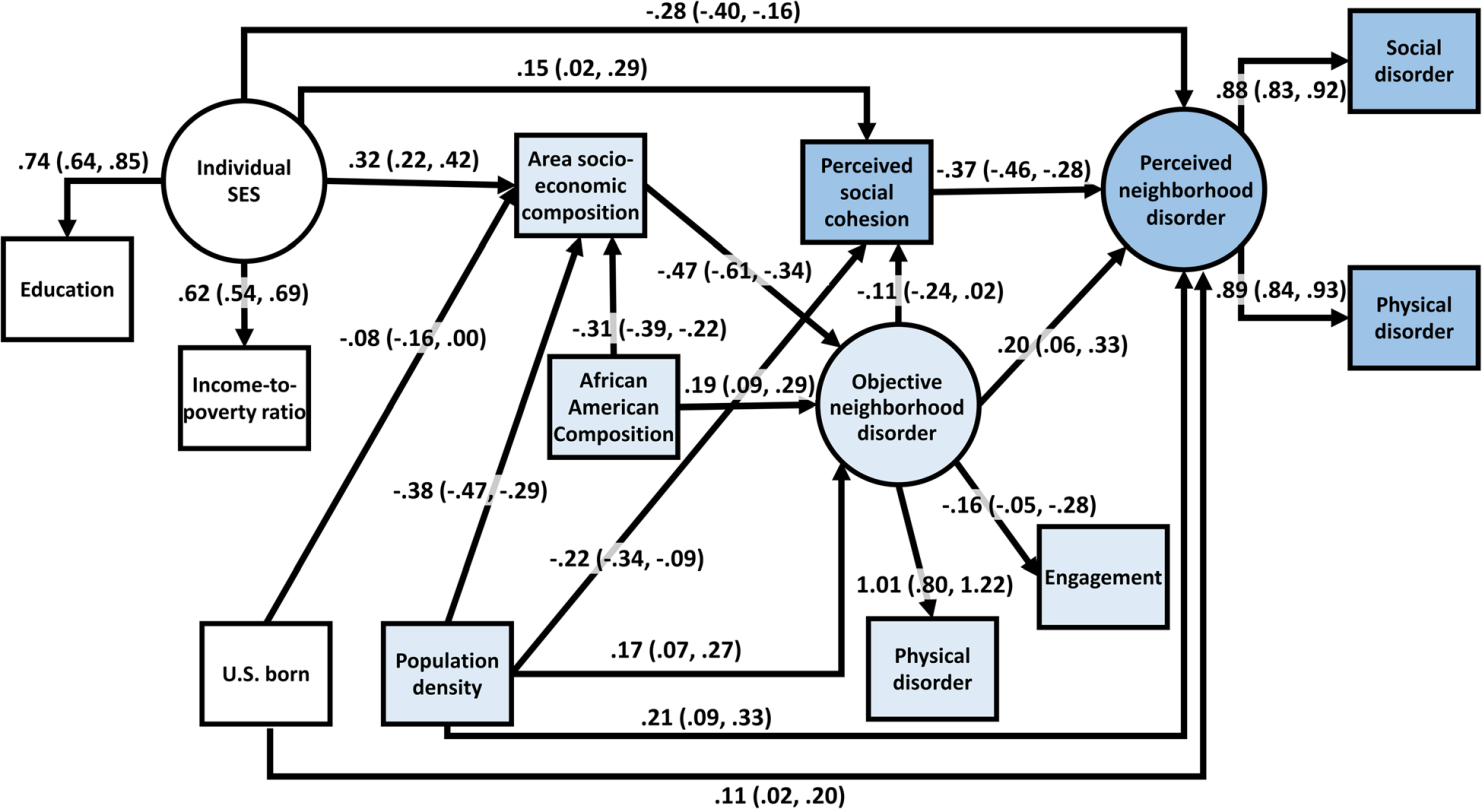
Final model, estimated associations between demographic, socioeconomic, perceived neighborhood, observed neighborhood, and census-based neighborhood factors, Women’s Circle of Health Follow-up Study, *n* = 310^1,2,3,4^. ^1^ Latent variables are circled and measured variables are in rectangles. Pathways between latent variables form the structural model and pathways involving measured variables form the measurement model. ^2^ Colorless shapes represent individual-level variables assessed 1 year prior to cancer diagnosis, light blue shapes represent neighborhood variables assessed at cancer diagnosis, medium blue shapes represent perceived neighborhood variables assessed two years following a cancer diagnosis. ^3^ Estimates are standardized path coefficients with 95% confidence intervals in parentheses. ^4^ Comparative fit index = 0.987, root mean square approximation = 0.043 (90% CI: 0.013, 0.068)

Model 1 (Fig. 1 without adjustment for potential confounders) yielded a comparative fit index of 0.983 and root mean square error approximation of 0.071 (90% CI: 0.035, 0.107), indicating questionable fit. Trimming pathways between variables with wide 95% CIs of standardized coefficients – omitting the pathway between the latent variables individual socioeconomic status and objective physical disorder, and the measured variables area socioeconomic composition and social cohesion – led to a model with similar fit; comparative fit index was 0.984 and root mean square error approximation was 0.062 (90% CI: 0.027, 0.096) (Model 2). Model 2 was expanded to allow for pathways between individual- and census-based demographic factors resulting in a model with adequate fit (Fig. 2); comparative fit index was 0.987 And root mean square error approximation was 0.043 (90% CI: 0.013, 0.068). In this final model, pathways between latent factors – individual SES and perceived neighborhood disorder and objective neighborhood disorder and perceived neighborhood disorder – form the structural model while all other pathways are part of the measurement model

For the structural model, higher perceived neighborhood disorder was associated with lower individual SES (β_standardized_ = − 0.28, 95% CI: − 0.40, − 0.16) and higher objective neighborhood disorder (β_standardized_ = 0.20, 95% CI: 0.06, 0.33). Perception of higher neighborhood disorder was associated with lower perceived neighborhood social cohesion (β_standardized_ = − 0.37, 95% CI: − 0.46, − 0.28), being US-born (β_standardized_ = 0.11, 95% CI: 0.02, 0.20), and higher population density (β_standardized_ = 0.21, 95% CI: 0.09, 0.33). Higher objective neighborhood disorder was associated with lower neighborhood socioeconomic composition (β_standardized_ = − 0.47, 95% CI: − 0.61, − 0.34), higher census tract African American/Black composition (β_standardized_ = 0.19, 95% CI: 0.09, 0.29), and higher population density (β_standardized_ = 0.21, 95% CI: 0.07, 0.27). Greater perceived neighborhood social cohesion was associated with higher individual SES (β_standardized_ = 0.15, 95% CI: 0.02, 0.29), lower population density (β_standardized_ = − 0.22, 95% CI: − 0.34, − 0.09), and lower objective neighborhood disorder (β_standardized_ = − 0.11, 95% CI: − 0.24, 0.02). Age, marital status, duration of neighborhood residence, or African American/Black segregation variables were not associated with any combinations of socioeconomic or neighborhood variables depicted in Fig. 1, and thus, did not meet criteria for inclusion in a final model. U.S. born, population density, and census tract African American/Black composition were not associated with any other variables besides those depicted in Fig. 2. The association between census tract African American/Black composition and perceived neighborhood disorder was close to zero with wide confidence intervals (β_standardized_ = − 0.00, 95% CI: − 0.12, 0.11). Table 3 summarizes hypothesized and modelled associations between key variables.

**Table 3:** Summary of hypothesized and modelled direct pathways between socioeconomic, perceived neighborhood, observed neighborhood, and census-based neighborhood socioeconomic factors, Women’s Circle of Health Follow-up Study, *n* = 310

		Negative or Positive Association	
**Dependent variable**	**Independent variable**	**Hypothesized**	**Modelled**
Perceived neighborhood disorder	Objective neighborhood disorder	Positive	Positive
	Perceived neighborhood social cohesion	Negative	Negative
	Area-level socioeconomic composition	Negative	No direct association
	Individual-level SES	Negative	Negative
Objective neighborhood disorder	Area-level socioeconomic composition	Negative	Negative
	Individual-level SES	Negative	No direct association
Perceived neighborhood social cohesion	Objective neighborhood disorder	Negative	Negative
	Area-level socioeconomic composition	Positive	No direct association
	Individual-level SES	Positive	Positive
Area-level socioeconomic composition	Individual-level SES	Positive	Positive

Results of the final model were nearly identical when restricting to participants who indicated no neighborhood moves within the 2 years prior to responding to the follow-up survey, with a slight attenuation of the association between objective neighborhood disorder and perceived neighborhood social cohesion (β_standardized_ = − 0.09, 95% CI: − 0.23, 0.04).

## Discussion

This study found support for specific pathways from audit- and census-based neighborhood variables to perceived neighborhood conditions among a longitudinal cohort of breast cancer survivors. Specifically, perception of greater neighborhood disorder 2 years following diagnosis was associated with lower perception of neighborhood social cohesion; greater objective neighborhood disorder, population density, and whether a participant was born in the US (vs. born in another country); and lower individual socioeconomic status within the year preceding diagnosis. Greater perceived neighborhood social cohesion was associated with higher individual socioeconomic status, lower population density, and lower objective neighborhood disorder. The latter association provides evidence of an indirect association from objective neighborhood disorder to perceived neighborhood disorder through perceived social cohesion. Moreover, area-level socioeconomic composition and African American/Black composition were not associated with perceived neighborhood disorder and individual socioeconomic status was not associated with objective neighborhood disorder within the final model.

Though this is the first known path analysis of these neighborhood and socioeconomic variables among Black breast cancer survivors, related studies have yielded similar findings. Early studies of perceived neighborhood disorder by Sampson and Raudenbush (2004) and Franzini et al. (2008) observed similar associations involving audited neighborhood disorder, individual socioeconomic status, and neighborhood social ties/exchanges (i.e., a construct related to social cohesion), which more recent studies have generally confirmed [51–53]. These previous studies also found mixed associations involving area-level African American/Black composition. The lack of an association between area-level African American/Black composition and perceived neighborhood disorder confirms results by Franzini et al. (2008) and Elo et al. (2009), both of which included study samples that were majority women and people identifying as African American/Black. Discrepancies between studies could be due to differences in study composition and sex- and race-specific relationships between African American/Black density and perceived disorder. Black and male respondents perceive lower neighborhood disorder compared to white and female respondents within similar levels of objective neighborhood disorder, and implicit cognitive biases based on racial identity, social norms, and familiarity of residence within specific neighborhood archetypes could influence variation in findings between neighborhood racial/ethnic composition and perceived disorder [6, 13, 51, 54, 55].

This study’s main contribution centers on the finding that residents’ perceptions of neighborhood disorder might be influenced by levels of objective, independently assessed neighborhood disorder directly as well as indirectly through the negative effects of objective disorder on perceptions of neighborhood social cohesion. Moreover, this study indicates that these relationships are independent of individual- and area-level socioeconomic and demographic factors. Earlier empirical studies and a recent review of perceived neighborhood disorder and health found a seemingly diminishing role for objective neighborhood disorder in favor of potentially more salient socioeconomic factors [2, 6, 13]. However, evidence from the longitudinal data herein suggests that objective neighborhood disorder might occupy a central, and influential position within pathways between socioeconomic factors and perceived neighborhood disorder. Motivated by results from recent experimental studies of objective disorder which demonstrate effects on health and well-being as well as ongoing, and frequent urban renewal projects which establish the feasibility of translation to practice [11, 56],interventions of objective neighborhood disorder might be one way to improve residents’ perceptions of a physically revitalized neighborhood with a favorable social environment. Unfortunately, our study could not test potential pathways between neighborhood factors and health behaviors and outcomes, or the preceding discriminatory practices and policies potentially related to all of the socioeconomic, neighborhood, and health constructs conceptualized herein (i.e., housing, criminal justice, mortgage lending, land use, urban policy, etc.) [15]. Future comprehensive longitudinal data collection should focus on these gaps.

Additional potential limitations include the data sources for measuring objective neighborhood disorder, lack of multilevel statistical modelling, lack of repeated measures to investigate trajectories and time-varying relationships, and ego-centric analysis of objective and perceived disorder. Image capture protocol behind Google Street View scenes is not widely known, image availability is spatio-temporally patterned, and a drawback especially relevant to this path analysis is temporal mismatch [38, 57]. Despite linking neighborhood perception and audit data by point-level address at diagnosis, the Google Street View image dates nearest to participants’ address might have been dated before or after diagnosis. Similarly, objective neighborhood disorder could have changed within neighborhoods over time such that neighborhood perceptions 2 years following diagnosis might be based on neighborhood characteristics that have changed from when women were diagnosed with cancer. The decision to not use multilevel statistical models is a limitation, but might have minimal impact on results because census tract cluster size was small (average 1.4 participant’s per census tract, 73.1% census tracts with only 1 participant) and variable inclusion in models was based on width of confidence intervals and model fit as opposed to a strict type-I error cut-off. It is unlikely that a multilevel model would lead to widening of confidence intervals large enough to change the final model [58]. Moreover, our results are similar to those of previous studies which employed multilevel modeling (without path analysis) [6, 13]. Use of address-level objective neighborhood disorder measures as opposed to an area-level measure estimated by aggregation could be a limitation, but was performed in other studies [13]. Moreover, there was little theoretical motivation to aggregate to some area-level that could be subject to additional sources of error such as the modifiable areal unit problem [59]. Studies that have performed aggregations have noted the high correlations between objective neighborhood disorder values within areas, similar to the spatial autocorrelation partial sill and range that we report [6, 51, 52]. Our methodology aligns with other studies involving observed and perceived neighborhood factors [13, 60].

Strengths of this study include the investigation of multiple neighborhood constructs and covariates potentially associated with health factors, temporal sequencing of covariates as conceptualized by previous literature, a moderately sized sample comprised of women self-identifying as African American/Black and previously diagnosed with breast cancer, investigation and finding of limited bias due to recent neighborhood moves, and use of objective neighborhood disorder measures with previously confirmed measurement properties [38, 47]. The uniqueness of this sample necessitates replication in samples with greater variability with respect to gender, racial-ethnic identity, geography, and health status.

## Conclusions

The inter-related nature of individual and area-level sociodemographic factors, perceived and objective neighborhood factors, and health behaviors and outcomes requires more careful attention to timing of measurement, mechanistic pathways, and covariate adjustment. Effective health-related interventions directed towards neighborhood environments can be better guided by future longitudinal studies that comprehensively assess numerous objective and perceived neighborhood constructs, demographic and socioeconomic factors at individual and area-levels, and health behaviors and outcomes. If replicated, the potential influence of objective neighborhood disorder on perceived neighborhood disorder directly and indirectly through reduced cohesiveness of neighborhoods can broaden options for interventions.

## Data Availability

The datasets generated and/or analysed during the current study are not publicly available in the form in which they were analyzed in this study due to individual privacy concerns.

## Abbreviations

SES: Socioeconomic status
WCHFS: Women’s Circle of Health Follow-up Study
CI: Confidence intervals
km: Kilometer
